# An Update on Ovarian Aging and Ovarian Reserve Tests

**DOI:** 10.22074/ijfs.2015.4591

**Published:** 2015-12-23

**Authors:** Ramazan Amanvermez, Migraci Tosun

**Affiliations:** 1 Department of Medical Biochemistry, Faculty of Medicine, Ondokuz Mayıs University, Samsun, Turkey; 2 Department of Obstetrics and Gynecology, Faculty of Medicine, Ondokuz Mayıs University, Samsun, Turkey

**Keywords:** Ovarian Aging, Infertility, Menopause, ROC Curve, Anti-Mullerian Hormone

## Abstract

Ovaries are the female organs that age more quickly than other tissues such as the uterus, the pituitary gland or pancreas. Different from males, an interesting question is why and how the females lose fertility so rapidly. During the aging process, both the number and quality of the oocytes in the ovaries decrease and reach to a point beyond that no more viable offspring may be produced and the associated cyclic endocrinological activities cease, entering the menopause in females at an average age of 50 years. Females who delayed childbearing with or without their willing until their 30 years or 40 years constitute the largest portion of the total infertility population. Ovarian reserve tests (ORTs) provide an indirect estimate of a female’s diminishing ovarian reserve or remaining follicular pool. This article briefly reviews recent progresses in relation to ovarian aging and ORTs.

## Introduction

**Fig.1:**
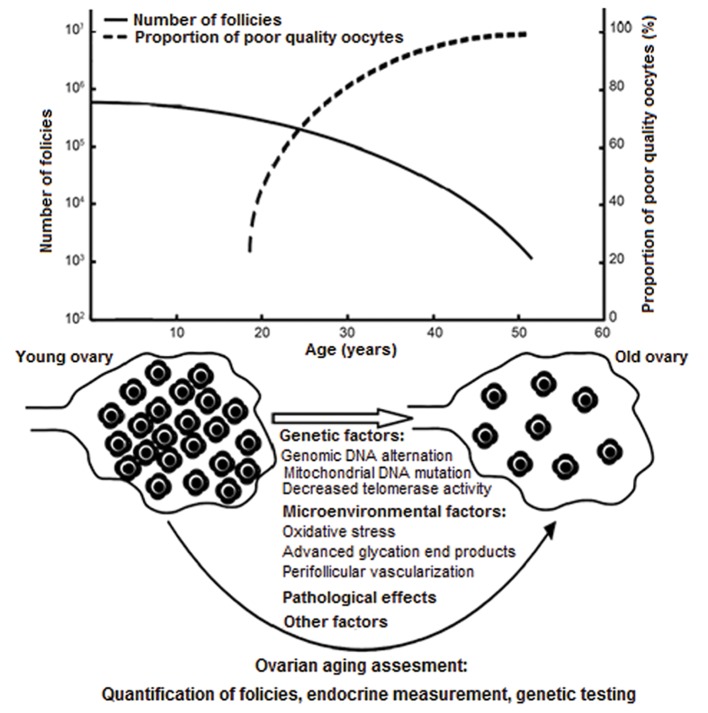
The number of primordial follicles in the ovaries and the quality of oocytes in relation to female age. Figure was represented by permission from Li et al. (3).

Physiologic ovarian aging is defined by age-specific declines of functional ovarian reserve within expected ranges. Scientific reports have long indicated that female ovarian reserve declines progressively with increasing chronological age. Fecundity, in both natural and stimulated ovarian cycles, reduces with maternal age that is why optimal fertility is accepted to be between 20 and 30 years old (1,3). In relation to this issue, the common concept of female reproductive aging assumes that the decline of reproductive potential or quantity and quality of oocyte/follicle pool estimates an agedependent loss of female fertility. Because ovaries undergo much more serious effects of aging than any other tissues of the female body, offspring has been demonstrated to be inversely proportional to age as shown in figure 1. 

Usually healthy female possesses ~400.000 primordial follicles at the beginning of puberty, each of which contains an immature ovum. About 300 to 400 follicles reach maturity during the reproductive life span of an adult female. The rest of the follicles are lost with apoptosis, which continue approximately for seven months during periods when there is no ovulation, such as pregnancy, breastfeeding or use of oral contraceptives. The most of oocytes are lost via apoptosis which is a more accelerated process in the last 10-15 years before menopause (4). The age-related decline of female fertility is frequently associated with the reduced monthly likelihood of conception and the increased probability that a pregnancy will terminate (e.g. the loss of embryo, pregnancy, fetal, and spontaneous abortion) sooner or later after conception or implantation between the ages of 35-45. In addition to these, scientific reports confirmed that the probability of achieving a pregnancy within one year was significantly higher in women <30 years than those in women <35 years (5,6). When a female reaches the mean age of 45, follicle pool usually decreases below a critical value of ~1000 or less follicles and irregular cyclic changes exist as the first clinical sign of ovarian aging (1,6). Along with these statements, female reproductive aging is nearly associated with a dysregulation of the gonadotrophin releasing hormone (GnRH) pulse generator in the hypothalamus due to a progressive lack of neuro-endocrine control from other brain parts, resulting in changes in the regular GnRH pulse pattern. The first sign of this change is the early elevation of follicle stimulating hormone (FSH) leading to acceleration of follicle depletion (1,6,7). Age-related changes in neuroendocrine response also contribute to the decline in reproductive function. There are two major theories on the origin of ovarian aging. One theory is that it is driven by the ovary itself. The rise of FSH is only secondary to loss of ovarian follicles and reduction of inhibin level. The other theory is that dysregulation of hypothalamic GnRH production, leading to rise in FSH levels and increase loss of follicles, causing ovarian aging. 

There is a large individual variability that exists in the age at which the various reproductive events occur in the context of normal reproductive female ovarian aging. In relation to issue, various gynecologic disorders or diseases and treatments, environmental and genetic factors contribute to biological ovarian aging and ovarian reserve decreasing. These factors include ovarian toxicants, cigarette smoking, alcohol abuse or chronic alcoholism, nutritional deficiencies, oxidative stress, some metabolic disorders, autoimmunity, long term stress-depression, iatrogenic treatments (pelvic surgeries, chemotherapy and radiotherapy), ovary inflammation and pelvic infection or tubal disease, severe endometriosis, meiotic division errors, chromosomal abnormalities, gene and mitochondrial DNA mutations in oocytes, and family history of early menopause in connection with the ovary aging and depletion of ovarian follicles and reduced ability to produce oocytes competent for fertilization and further development as well as infertility (3,8,15). 

The probability of spontaneous conception (fecundity) and infertility treatment success depend on functional ovarian reserve particularly and total ovarian reserve. As both parameters decline with advancing age, pregnancy chances, thereupon, decrease in parallel (16,26). Age is obviously known to be the most important factor determining the pregnancy potential in normally cycling females. Therefore, chronological age alone has a limited value in predicting individual responses. Age is the main determinant of the chance of successful pregnancy. Moreover, the quantitative response to ovarian stimulation with gonadotrophins depends on the ovarian reserve. This depends on age, genetic and some environmental factors. It means that 95% of reproductive aging is determined by age and genetics, and less than 5% is determined by environmental factors (6,27). The decline rate of ovarian reserve varies among females, making it a challenge to estimate an individual female’s remaining reproductive function. Therefore, it is necessary to evaluate females’ reproductive potential prior to infertility treatment in order to succeed (11). Hence, ultrasonography, various biochemical and histo-pathological markers (tests) have become popular in the last years in assessment of ovarian reserve. The aim of these tests is to contribute to counseling and treatment of infertile couples (1,2,17,28). 

### Ovarian reserve tests (ORTs)

#### Anti-mullerian hormone (AMH)

AMH is a dimeric glycoprotein expressed by granulose cells of pre-antral and early antral follicles of ovary during the female reproductive life span. AMH level in blood is considerably low before puberty, but after puberty, it reaches a maximum level and then its concentration progressively declines as a sign of exhaustion of total follicular reserve throughout reproductive life, reaching undetectable values by menopause (14,18). Studies have shown that AMH is a better marker than antral follicle count (AFC), baseline FSH, estradiol (E_2_) and inhibin B in estimating ovarian reserve. Age-related decrease in the number of oocytes leads to a decrease in E_2_ and inhibin B levels, as a result of which FSH rises. In addition, AMH levels correlate strictly with AFC and age. Recent reports have indicated that AMH, indeed, can be used as a test for ovarian aging and reserve as it can be measured in blood during any phase of the menstrual cycle (17,20). AMH appears to be the best biochemical-endocrine marker in assessing the age-related decline of the ovarian pool and predicting the ovarian response of induced patients including poor and hyper-responses of *in vitro* fertilization (21). 

#### Antral follicle count

Ovarian volume and AFC, evaluated by transvaginal ultrasonography, provide direct ovarian assessments. The latter, defined as the number of follicles smaller than 10 mm in diameter in the early follicular phase, is considered to have the best discriminating potential for a poor ovarian response compared to the total ovarian volume and basal serum values of FSH, E_2_, and inhibin B on 3^rd^ day of the cycle. Therefore, it is accepted to be predictor of the number of oocytes remaining. AFC also reflects the ovarian reserve better than ovarian volume in infertile patiens(2,14,17). 

#### Basal follicle stimulating hormone

Elevation in basal FSH level, occurring usually at the ages of 35-40, is the first sign of ovarian aging that can be detected in females. Early follicular phase (basal) or cycle day-3 FSH level is an indirect marker of ovarian reserve and reflects the negative feedback effects of inhibin-B and E_2_ on hypophysis (2,4,14). An increase in blood FSH levels occurs due to follicle depletion. In females with regular cycling, very high FSH levels may predict a poor response, thus this can be useful in screening of a small infertile group. Along with other markers, it may be used to counsel families about poor response (2). 

#### Basal estradiol

Early rise in blood E_2_ (17β-estradiol is derived almost exclusively from the ovaries, and its measurement is frequently considered sufficient to evaluate ovarian function) level is known as a consequence of the advanced follicular development and early selection of a dominant follicle observed in cycling females with increased FSH levels (22). 

A combination of FSH and E_2_ in screening for declined ovarian reserve seems to be more sensitive than either test alone. However, basal E_2_ level has little value as an ovarian reserve test and its routine use is not advised (2,17). 

#### Inhibin B

Inhibin B is a heterodimeric glycoprotein released by the granulose cells of the follicles, and its concentration peaks during the follicular phase. The inhibin B level has been used in conjunction with serum FSH and E_2_ to assess ovarian function. A decline in inhibin B concentrations in early follicular phase may be observed before an increase in FSH level. Inhibin B seems to be a good indicator of ovarian activity, whereas it has minimal value in predicting ovarian reserve, so that its routine use is not recommended (1,2,17). 

#### Gonadotrophin releasing hormone agonist stimulation test (GAST)

GAST is associated with the assessment of serum E_2_on days 2-3 of the cycle following subcutaneous application of 100 μg GnRH agonist (e.g. Triptorelin). The response of E_2_ to GnRH agonist is an indirect indicator of ovarian reserve. As GnRH agonists may lead to decrease E_2_ elevation when the follicular cohort is small in ovarian tissue; an increase in serum E_2_ is considered to be indicative of good ovarian function. Although this test seems to be valuable in prediction of poor ovarian reserve, it is not superior to AMH, AFC or inhibin B in this theme (2,14,17). 

#### Exogenous follicle stimulating hormone ovarian reserve test (EFORT)

EFORT involves the measurement of basal FSH and E_2_ following the administration of 300 IU FSH on the 3^rd^ day of the menstrual cycle. The change in basal FSH and a rise in E_2_ levels (>30 pg/ml) 24 hours after FSH administration, may predict ovarian reserve. However, the authors did not recommend this test alone for identification of hyperresponders in assisted reproductive technologies (ART) cycles (2, 14, 17).

#### Clomiphene citrate challenge test (CCCT)

CCCT is a provocative test aimed to assess ovarian reserve. In this test, 100 mg CC is administrated daily from day 5 to day 9 of the cycle. Day- 3 FSH and E_2_ levels are measured and followed by the administration of CC from day 5 to day 9. FSH-E_2_ measurements are repeated on day 10 and high day-10 FSH level suggests poor ovarian reserve. CCCT effectively reflects the quantity and quality of the recruited oocytes, but its predictive value is low, while it is expensive and more time consuming. Also, meta-analysis has reported that CCCT is not better than basal FSH in predicting a clinical pregnancy (2,17,23). 

#### Ovarian biopsy

Studies on ovarian biopsy which is done at laparotomy or laparoscopy have indicated that follicular density declines with age and is correlated with the ovarian volume in female <35 years. However, the distribution of follicles is not uniform within the ovary, so that the biopsy is not able to represent the true follicular density. Therefore, ovarian biopsy is rarely necessary and it is not recommended as an ORT (2,17). 

#### Genetic markers of ovarian reserve

With developments in molecular genetics has given hope to researchers about prediction of single nucleotide polymorphisms (SNPs) in gonadotropins and their receptor genes, *BMP-15, GDF9, FMR1* gene, *MCM8* gene, and the other candidate genes which identify females with a genetic predisposition to early ovarian aging (6,24,25). There are currently no reliable genetic markers of ovarian reserve that can be used as a routine test (screening/diagnostic). 

## Conclusion

**Fig.2:**
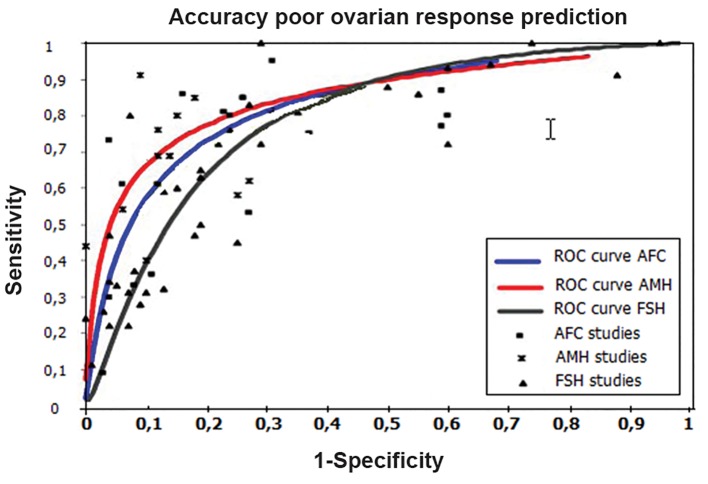
ROC curves of studies reporting on the performance of the AFC, AMH and basal FSH tests to predict poor ovarian response (figure was produced by data of references 1, 2, 17). ROC; Receiver operating characteristic curve, AFC; Antral follicle count, AMH; Anti-mullerian hormone and FSH; Follicle stimulating hormone.

The current literature reports and meta-analysis ROC curves indicate that AMH and AFC are currently promising predictor tests, and FSH is a screening test widely used for assessment of diminished ovarian reserve in addition to chronological age. AMH can be applied to all females to identify decreased ovarian reserve before it reaches a critical low value (Fig .2) (1,26,28). 

Increasing female education and their career levels, and participation in the labor force are important trends in freedom of females currently taking place in most countries including Turkey. A farseeing of this societal adjustment involves extraordinary changes in reproductive behavior such as consciously choosing a life without children and delays in childbearing. A number of females also decide not to have a child at younger ages, but change their minds at later ages. It is known that some tissue/cell functions and aging are different in females and males. Though oocytes/germ cells are not absolutely required for the living, reproduction is critically important for the survival of the population of the living species in the world. A healthy baby should be a nice heritage for families, so it may be suggested to females to deliver a baby or babies before 30 years because of the possible ovarian aging. 
